# Factors influencing peripheral immunity and its links to brain disorders

**DOI:** 10.1038/s41398-024-02963-3

**Published:** 2024-06-05

**Authors:** Brendan Jen-Wei Tan, Ling-Ling Chan, Eng-King Tan, Bin Xiao

**Affiliations:** 1 Department of Neurology, National Neuroscience Institute, Singapore, Singapore; 2 Duke-NUS Medical School, Singapore, Singapore

**Keywords:** Pathogenesis, Diseases

Dear editor,

We read with interest the study by Zhong et al. [1], which studies the association between peripheral immunity and risk of incident brain disorders in a prospective cohort study of >160,000 participants from the UK Biobank. They found evidence of elevated peripheral innate immunity markers associated with an increased risk of neurological and psychiatric disorders. Interestingly, their findings of abnormal innate immunity are not specific to any neurological conditions. The authors took into account various factors in the analysis, including age, gender, education, ethnicity, and blood pressure. In addition, they excluded diseases that may influence peripheral immune cells. While we recognise the difficulty in excluding all possible confounding variables, there are several common factors that have not been considered in the analysis and not highlighted in the discussion.

First, several intrinsic and extrinsic factors affect peripheral blood counts and cytokines, including smoking, alcohol, caffeine, diet and physical activity among others. A study of 35,000 healthy men and women has shown that the range of white cell count for smokers is different from that of non-smokers, and this is further affected by smoking intensity. There is also an association between white cell count and several variables, including haemoglobin, cholesterol, uric acid, blood sugar, etc. [2]. It would be interesting if Zhong et al. had included smoking and smoking intensity as covariates in their analysis.

In another cross-sectional study of 5000 Japanese males, after controlling for covariates, such as age, body mass index, lipids, fasting glucose and blood pressure, the white count was associated with alcohol consumption in non-smokers and smokers [3]. The effect of diet on white cell counts and other innate periphery markers is also another factor to consider. For example, in a study of 986 healthy subjects, there was a significant correlation between elevated levels of white blood cells, lymphocytes and basophils with lower habitual intake of vegetables. The intake of vegetables accounted for up to 6.5% of variation in white blood cells. Interestingly, certain bacteria species-mediated some of the effects of vegetable intake on lymphocyte counts. The white cell counts also correlated with specific inflammatory markers, suggesting a link between diet and inflammation [4].

Prevalent among the general population, caffeine consumption is recognized as a practice that may have a modulating effect on the risk of chronic neurological conditions [5]. There have been suggestions that physical activity can promote an elevation of packed cell volume that this could be facilitated by caffeine. In a study on caffeine effects on white blood cells during exercise, investigators have demonstrated that caffeine exercise increased blood lymphocyte count by 38% and this could be enhanced by another 35% when combined with caffeine. Caffeine intake facilitated a rise in circulating monocytes (independent of exercise), and both exercise and caffeine seemed to have additive effects on segmented neutrophils [6]. Separately a study that examined the effect of the amount of aerobic exercise on total white blood cell and its subfraction counts in overweight postmenopausal women found that exercise was able to reduce the neutrophil and total white cell count in a dose-dependent manner [7].

In summary, smoking, alcohol and caffeine consumption and physical activity are some of the major factors that could be considered to strengthen the work of Zhong et al. in their study of peripheral immunity. Importantly, these factors are also directly linked to various neurological conditions. In addition, the potential effect of polypharmacy on peripheral innate immunity markers is an area that could be explored. The real-time temporal relationship between peripheral immunity markers (fluctuations, rate of change etc.) and incident neurological disorders can only be properly addressed in a prospective longitudinal study with a systematic evaluation of standardized outcome measures. Nevertheless, Zhong et al.’s study provides impetus for further studies on the role of peripheral immunity markers as potential biomarkers of chronic neurological disease progression and prognosis.
